# Evaluation of the calcaneo-stop procedure with FIT-CS for the surgical treatment of juvenile flatfoot

**DOI:** 10.1007/s00264-026-07030-8

**Published:** 2026-09-16

**Authors:** Gianluca Testa, Marco Sapienza, Michela Marchetti, Emanuele Perricone, Marco Simone Vaccalluzzo, Martina Ilardo, Alessia Caldaci, Federico Canavese, Andrea Vescio, Vito Pavone

**Affiliations:** 1 Department of General Surgery and Medical Surgery Specialties, Section of Orthopaedics, A.O.U. Policlinico Rodolico- San Marco, University of Catania, Catania, Italy; 2 Ortopaedica and Traumatology Department, IRCCS Istituto Giannina Gaslini, University of Genoa, 16147 Genoa, Italy; 3 Department of Life Science, Health, and Health Professions, Link Campus University, 00165 Rome, Italy

**Keywords:** Paediatric flexible flatfoot, Calcaneo-stop arthroereisis, Subtalar arthroereisis, Foot deformity, Functional outcomes, Radiographic outcomes

## Abstract

**Purpose:**

Flexible flatfoot is one of the most common paediatric foot deformities. Although most cases remain asymptomatic, a subset of patients develop persistent pain and functional limitations requiring surgical treatment. This study evaluated the medium-term clinical, functional, and radiographic outcomes of Calcaneo-stop arthroereisis in children with symptomatic flexible flatfoot.

**Methods:**

A retrospective analysis of prospectively collected data was performed on consecutive pediatric patients who underwent Calcaneo-stop arthroereisis between January 2018 and December 2022. Clinical outcomes were assessed using the American Orthopaedic Foot and Ankle Society (AOFAS) Hindfoot Score, the Oxford Ankle Foot Questionnaire for Children (OxAFQ-C), and the Catania Flatfoot Score (CTF). Radiographic outcomes included Kite's and Costa-Bertani angles. Patients were evaluated preoperatively and at one and three year follow-up. Repeated-measures ANOVA with Bonferroni-adjusted post-hoc comparisons was used.

**Results:**

A total of 121 consecutive patients (218 treated feet) completed a minimum follow-up of three years. The AOFAS score improved from 44.99 ± 10.59 preoperatively to 92.46 ± 8.78 at final follow-up (*p* < 0.001). Similar improvements were observed for the OxAFQ-C and CTF scores, together with significant correction of Kite's angle (30.38° to 24.02°) and Costa-Bertani angle (155.95° to 123.22°) (all *p* < 0.001). No significant differences were observed between the first and third postoperative year. The overall complication rate was 7.4%, with only two patients requiring revision surgery because of implant screw breakage.

**Conclusion:**

Calcaneo-stop arthroereisis provided significant and sustained clinical, functional, and radiographic improvement at a minimum follow-up of three years. Clinical and radiographic correction remained stable after the first postoperative year, while the procedure demonstrated a low complication rate, supporting its use as a safe and effective surgical option for carefully selected pediatric patients.

## Introduction

Flexible flatfoot is one of the most common foot deformities encountered in paediatric orthopaedic practice and is characterized by collapse of the medial longitudinal arch, hindfoot valgus, and excessive subtalar pronation, resulting in altered foot biomechanics and, in some patients, pain and functional impairment [1]. During early childhood, flexible flatfoot is generally considered a physiological condition because the medial longitudinal arch develops progressively with growth. Consequently, its prevalence decreases with age as spontaneous correction occurs in the majority of children [1–3]. Although most cases remain asymptomatic and do not require treatment, a subset of patients develops persistent pain, gait abnormalities, fatigue, or limitations in sports and daily activities despite appropriate conservative management [1–8].

When conservative treatment fails, several surgical procedures have been proposed to restore physiological hindfoot alignment and improve foot function. Among these, subtalar arthroereisis using the Calcaneo-stop technique has gained increasing popularity because it is minimally invasive, technically straightforward, and capable of correcting excessive hindfoot pronation while preserving joint anatomy [4, 8]. The procedure combines a mechanical restraint to excessive subtalar motion with a proprioceptive effect that may promote progressive neuromuscular adaptation during skeletal growth, thereby contributing to maintenance of the achieved correction [8–12].

Despite the widespread clinical use of Calcaneo-stop arthroereisis, several aspects remain incompletely defined, including the durability of clinical and radiographic correction during medium-term follow-up and the identification of factors potentially associated with treatment success or complications. Furthermore, although several studies have reported favorable outcomes, differences in study design, outcome measures, and follow-up duration make direct comparison between published series challenging [2, 4, 7, 10–14].

Therefore, the aim of the present study was to evaluate the medium-term clinical, functional, and radiographic outcomes of Calcaneo-stop arthroereisis using the FIT-CS screw in a consecutive cohort of paediatric patients with symptomatic flexible flatfoot through a retrospective analysis of prospectively collected data. Clinical outcomes were assessed using validated functional scores, whereas radiographic correction was evaluated through standardized measurements of Kite's angle and Costa-Bertani angle.

The research question of this study was whether the Calcaneo-stop procedure using the FIT-CS screw provides significant and sustained clinical, functional, and radiographic improvement in children with symptomatic flexible flatfoot.

The primary endpoint was the change in the American Orthopaedic Foot and Ankle Society (AOFAS) Hindfoot Score from baseline to the three year follow-up. Secondary endpoints included changes in the Oxford Ankle Foot Questionnaire for Children (OxAFQ-C), the Catania Flatfoot Score (CTF), Kite's angle, Costa-Bertani angle and postoperative complications.

We hypothesized that the Calcaneo-stop procedure would result in significant improvements in all clinical, functional, and radiographic outcomes, which would be maintained throughout the follow-up period.

## Material and methods

### Study design

This monocentric observational retro-prospective study was conducted at the Department of Orthopaedics and Traumatology, University Hospital "G. Rodolico–San Marco", Catania, Italy, following approval by the Institutional Review Board (Protocol No. 03/2026/PAR; February 18, 2026). The approved study protocol includes a retrospective component (January 2018–December 2025) and a prospective component (January 2025–December 2027).

The present study represents a retrospective analysis of prospectively collected clinical and radiographic data and includes consecutive paediatric patients who underwent Calcaneo-stop arthroereisis between January 2018 and December 2022. Only patients who completed a minimum clinical and radiographic follow-up of three years were included in the present analysis.

### Patients

**Fig. 1:**
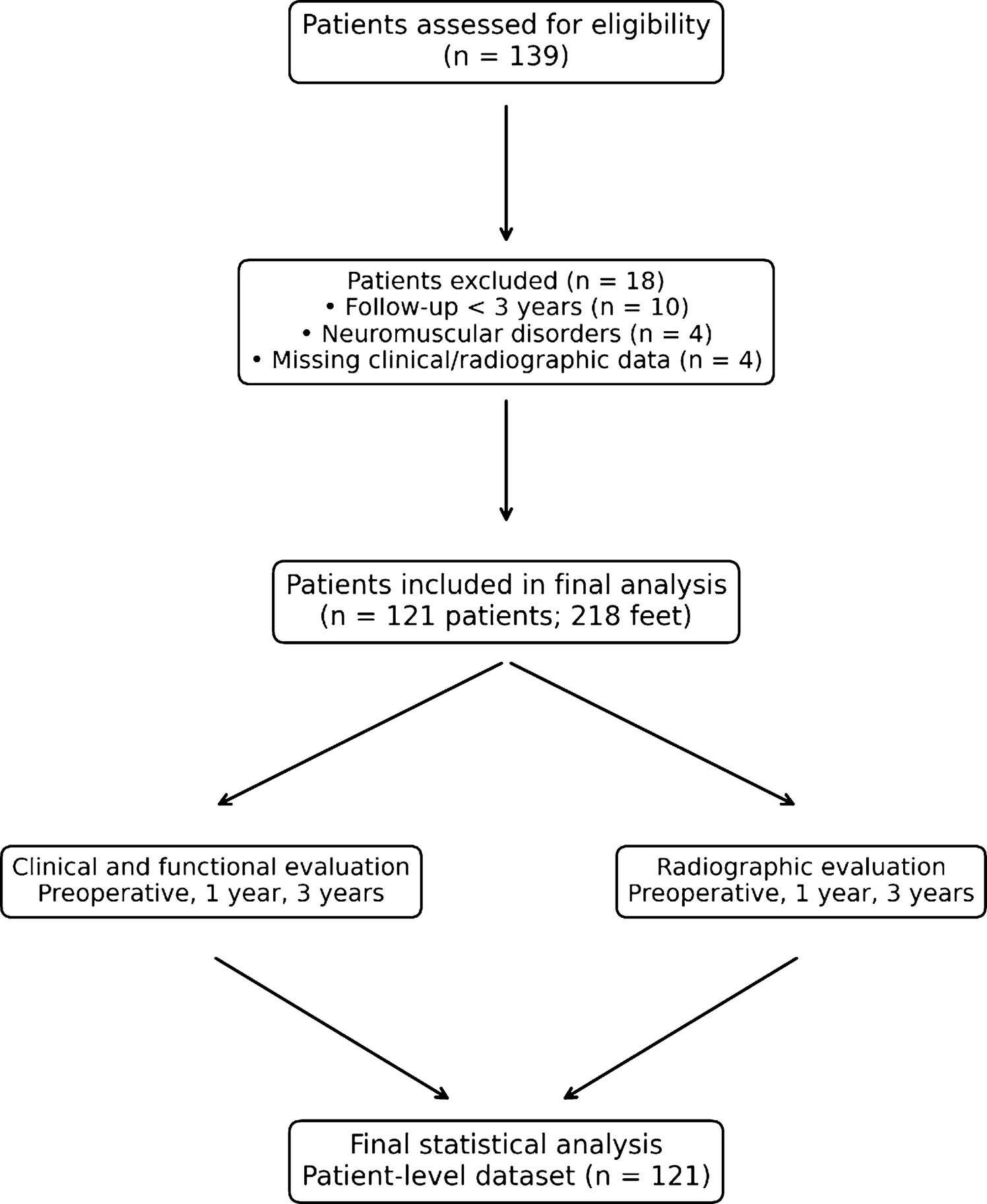
STROBE flow diagram illustrating patient selection, exclusion criteria, follow-up completion, and inclusion in the final statistical analysis

Between January 2018 and December 2022, 139 consecutive paediatric patients with symptomatic flexible flatfoot were assessed for eligibility. Following application of the predefined inclusion and exclusion criteria, 121 patients (218 feet) fulfilled the eligibility criteria and were included in the final analysis (Fig. 1). Eighteen patients were excluded, including ten because they had not completed the minimum follow-up of three years, four due to neuromuscular disorders, and four because of incomplete clinical or radiographic data.

#### Inclusion criteria

Patients were eligible if they met all of the following criteria:

- age between eight and 14 years at the time of surgery;
- symptomatic idiopathic flexible flatfoot confirmed by clinical and radiographic evaluation;
- persistence of symptoms despite at least six months of conservative treatment, including orthotic insoles, stretching exercises, and physical therapy;
- pain or functional limitation affecting daily or sports activities;
- availability to complete the postoperative follow-up protocol.

#### Exclusion criteria

Patients were excluded in the presence of:

- rigid flatfoot;
- congenital foot deformities or structural skeletal abnormalities;
- neurological or neuromuscular disorders;
- previous surgical procedures involving the foot or lower limb;
- incomplete clinical or radiographic data;
- failure to complete the minimum three-year follow-up.

### Outcome measures

The primary endpoint of the study was the improvement in functional outcome following Calcaneo-stop arthroereisis, assessed using the American Orthopaedic Foot and Ankle Society (AOFAS) Hindfoot Score.

Secondary endpoints included:

- Oxford Ankle Foot Questionnaire for Children (OxAFQ-C);
- Catania Flatfoot Score (CTF);
- radiographic correction assessed by Kite's angle and Costa-Bertani angle;
- postoperative complications.

Clinical and radiographic evaluations were performed preoperatively and at one year and three year follow-up according to the institutional follow-up protocol.

Clinical outcomes were assessed using the American Orthopaedic Foot and Ankle Society (AOFAS) Hindfoot Score, which combines physician-based assessment of alignment with patient-reported pain and function, the Oxford Ankle Foot Questionnaire for Children (OxAFQ-C), which evaluates physical, emotional, school, and social domains, and the Catania Flatfoot Score (CTF), an objective clinical scoring system specifically developed for paediatric flexible flatfoot.

Standardized weight-bearing anteroposterior and lateral foot radiographs were obtained preoperatively and at each postoperative evaluation. Radiographic assessment included measurement of Kite's angle on the anteroposterior view and the Costa-Bertani angle on the lateral view according to previously described methods. All radiographic measurements were independently performed by three experienced orthopaedic surgeons using standardized measurement techniques.

### Surgical technique

All procedures were performed using the Calcaneo-stop technique under general or regional anaesthesia with the patient in the supine position.

A lateral skin incision of approximately 1 cm was made over the sinus tarsi. Under fluoroscopic guidance, a guidewire was introduced through the calcaneal notch, followed by insertion of a cannulated self-drilling and self-tapping FIT-CS® screw (Ge.Me.S., Italy) in an anteromedial direction. During screw insertion, a forced inversion manoeuvre was performed to restore physiological talocalcaneal alignment and reduce talocalcaneal divergence.

The implant acts as both a mechanical restraint limiting excessive subtalar pronation and a proprioceptive stimulus that promotes progressive neuromuscular adaptation during growth. After satisfactory correction had been achieved under fluoroscopic control, the wound was closed with absorbable sutures and a compressive dressing was applied.

According to our institutional protocol, elective implant removal may be considered approximately three to four years after surgery when clinically indicated. Implant removal, however, was not considered an outcome measure of the present study.

### Statistical analysis

Statistical analyses were performed using IBM SPSS Statistics for Windows, Version 27.0 (IBM Corp., Armonk, NY, USA).

Continuous variables are presented as mean ± standard deviation (SD), whereas categorical variables are expressed as frequencies and percentages.

Data normality was assessed using the Shapiro–Wilk test.

For all statistical analyses, the patient represented the statistical unit of analysis. In patients undergoing bilateral surgery, radiographic measurements obtained from both feet were averaged to generate a single patient-level value, thereby avoiding pseudoreplication and unit-of-analysis bias.

Changes in clinical and radiographic outcomes over time were evaluated using repeated-measures analysis of variance (RM-ANOVA). When a significant overall time effect was observed, pairwise comparisons between time points were performed using Bonferroni-adjusted post-hoc tests.

Exploratory multivariable regression analyses were performed to investigate the potential influence of age, sex, bilateral involvement, and baseline radiographic severity on postoperative clinical improvement.

A two-sided *p* value < 0.05 was considered statistically significant.

## Results

### Study population

Between January 2018 and December 2022, 139 consecutive patients were assessed for eligibility. After application of the predefined inclusion and exclusion criteria, 121 patients (218 feet) were included in the final analysis (Fig. 1). Eighteen patients were excluded, including ten because they had not completed the minimum follow-up of three years, four due to neuromuscular disorders, and four because of incomplete clinical or radiographic data.

The final cohort comprised 85 males (70.25%) and 36 females (29.75%), with a mean age at surgery of 11.49 ± 1.30 years (range, 8–14 years). Bilateral surgery was performed in 97 patients (80.17%), whereas 24 patients (19.83%) underwent unilateral procedures (14 right and 10 left feet). The mean follow-up was 49.5 ± 7.8 months.

Baseline demographic and clinical characteristics are summarized in Table 1.

**Table 1:** Baseline characteristics of the study population

Variable	Value
Patients assessed for eligibility	139
Patients included in the final analysis	121
Excluded patients	18
Follow-up < 3 years	10
Neuromuscular disorders	4
Missing clinical/radiographic data	4
Treated flatfeet	218
Male	85 (70.25%)
Female	36 (29.75%)
Mean age at surgery (years)	11.49 ± 1.30
Age range (years)	8–14
Bilateral procedures	97 (80.17%)
Unilateral procedures	24 (19.83%)
Right	14
Left	10
Mean follow-up (months)	49.5 ± 7.8

Values are presented as mean ± standard deviation (SD) or number (%)

### Clinical and radiographic outcomes

All clinical, functional, and radiographic outcome measures demonstrated significant improvement following Calcaneo-stop arthroereisis (Table 2).

**Table 2:** Clinical and radiographic outcomes following Calcaneo-stop arthroereisis

Outcome	Preoperative Mean ± SD	1-year Mean ± SD	3-year Mean ± SD	Overall p (RM-ANOVA)	Pre vs 1 year	1 year vs 3 years	Pre vs 3 years
AOFAS score	44.99 ± 10.59	90.32 ± 9.88	92.46 ± 8.78	< 0.001	< 0.001	NS	< 0.001
OxAFQ-C score	22.04 ± 3.32	40.64 ± 3.51	41.76 ± 3.62	< 0.001	< 0.001	NS	< 0.001
Catania Flatfoot Score	90.59 ± 5.40	29.33 ± 7.74	28.27 ± 7.42	< 0.001	< 0.001	NS	< 0.001
Kite's angle (°)	30.38 ± 1.35	23.89 ± 1.40	24.02 ± 1.40	< 0.001	< 0.001	NS	< 0.001
Costa-Bertani angle (°)	155.95 ± 4.39	122.94 ± 1.46	123.22 ± 1.62	< 0.001	< 0.001	NS	< 0.001

RM-ANOVA = repeated-measures analysis of variance. NS = not statistically significant after Bonferroni correction

Repeated-measures ANOVA revealed a statistically significant effect of time for all evaluated clinical and radiographic parameters (all *p* < 0.001). Bonferroni-adjusted post-hoc analyses confirmed significant improvements between the preoperative assessment and both postoperative evaluations (all *p* < 0.001), whereas no statistically significant differences were observed between the one year and three year follow-up assessments, indicating maintenance of the achieved correction and functional recovery over time.

**Fig. 2:**
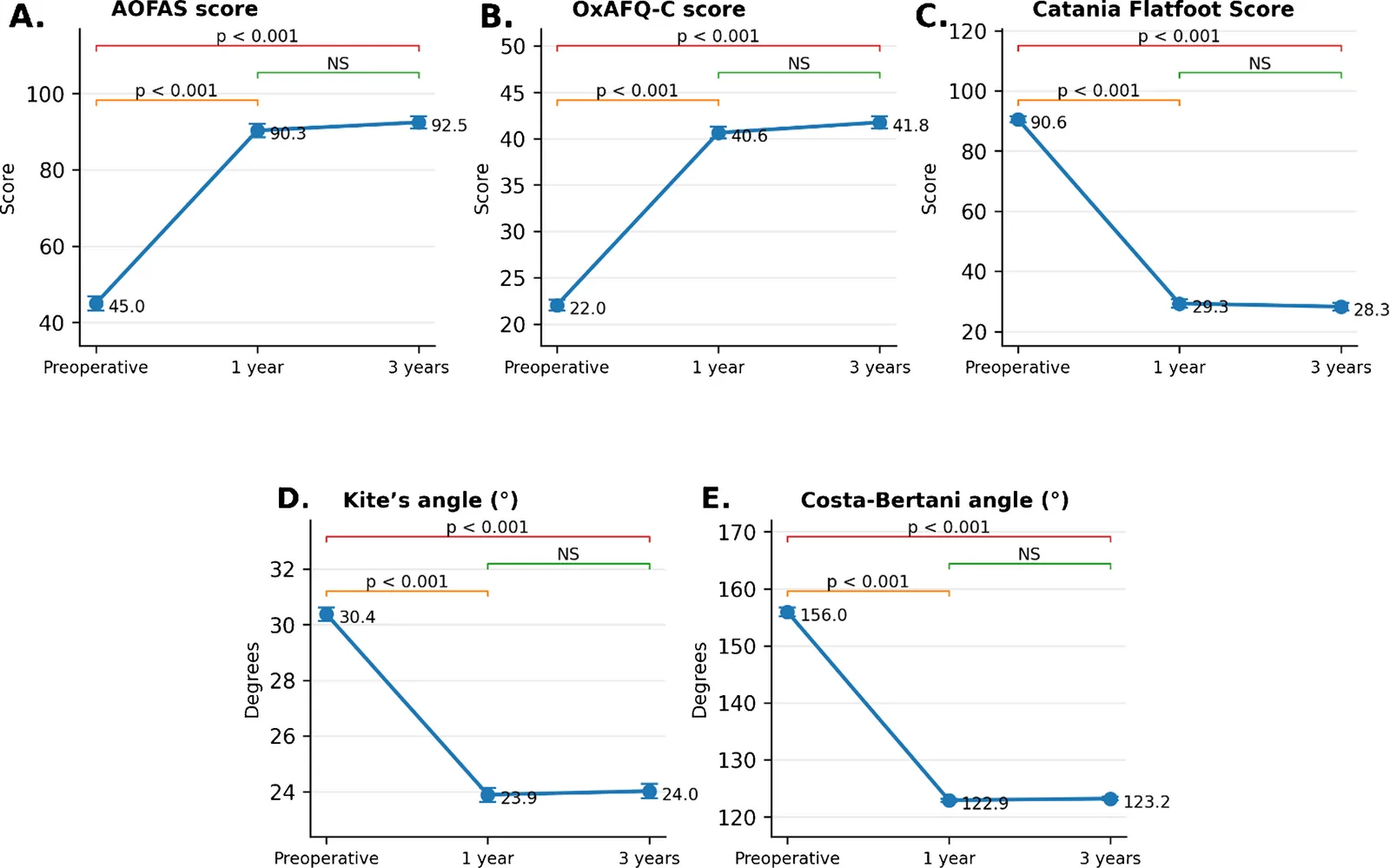
Evolution of clinical, functional, and radiographic outcomes following Calcaneo-stop arthroereisis: **A**) AOFAS score; **B**) OxAFQ-C score; **C**) Catania Flatfoot Score; **D**) Kite's angle; **E**) Costa-Bertani angle. Values are presented as mean with 95% confidence intervals. Repeated-measures ANOVA followed by Bonferroni-adjusted post-hoc comparisons showed significant improvements between preoperative values and both postoperative assessments, whereas no significant differences were observed between 1-year and 3-year follow-up. NS, not significant

Mean values for all evaluated outcomes are reported in Table 2, while their temporal evolution is illustrated in Fig. 2.

### Exploratory regression

Exploratory multivariable regression analyses were performed to evaluate whether age, sex, bilateral involvement, or baseline radiographic severity independently influenced postoperative clinical improvement. None of the investigated variables emerged as a significant independent predictor of the postoperative outcomes (all *p* > 0.05). Therefore, no clinically meaningful predictors of treatment response were identified in the present cohort.

### Postoperative complications

Overall, nine postoperative complications (7.4%) were recorded during the follow-up period (Table 3).

**Table 3:** Postoperative complications

Complication	Patients n (%)	Management	Outcome
Superficial wound erythema	7 (5.8%)	Conservative treatment	Complete resolution
Implant screw breakage	2 (1.7%)	Revision surgery	Successful revision
Deep infection	0	—	—
Neurovascular injury	0	—	—
Implant loosening	0	—	—
Overcorrection	0	—	—
Undercorrection	0	—	—
**Total complications**	**9 (7.4%)**		

Seven patients (5.8%) developed mild superficial wound erythema around the surgical incision, which resolved completely following conservative treatment without affecting the final clinical outcome. Two patients (1.7%) experienced implant screw breakage approximately five months after surgery and underwent successful revision surgery.

No cases of deep infection, neurovascular injury, implant loosening, overcorrection, or undercorrection were observed.

Routine implant removal was not considered an outcome measure of the present study because the predefined follow-up protocol was specifically designed to evaluate medium-term clinical and radiographic outcomes rather than elective screw removal.

## Discussion

The principal finding of the present study is that Calcaneo-stop arthroereisis using the FIT-CS screw resulted in significant clinical, functional, and radiographic improvement in children with symptomatic flexible flatfoot, with correction remaining stable throughout the three-year follow-up period. Furthermore, the procedure was associated with a low complication rate, supporting its role as a safe and reliable surgical option in appropriately selected paediatric patients.

Flexible flatfoot represents one of the most frequent foot deformities in children. Although most cases remain physiological and asymptomatic during growth, a proportion of patients develop persistent pain, functional impairment, gait abnormalities, and limitations in sports or daily activities despite appropriate conservative management [1, 9]. In these patients, the primary objective of surgical treatment is to restore physiological hindfoot alignment while improving function and quality of life. Among the available surgical techniques, Calcaneo-stop arthroereisis has progressively gained widespread acceptance because of its minimally invasive nature, technical simplicity, and preservation of subtalar joint anatomy [8–12].

The present study demonstrated significant improvements in all evaluated clinical outcome measures, including the AOFAS Hindfoot Score, the Oxford Ankle Foot Questionnaire for Children, and the Catania Flatfoot Score. More importantly, these improvements were maintained between the first and third postoperative year, suggesting that the functional benefits achieved after surgery remain stable during medium-term follow-up. Our findings are consistent with previous clinical series reporting favourable functional outcomes following Calcaneo-stop arthroereisis [2, 3, 11, 12]. Similarly, recent systematic reviews have confirmed that subtalar arthroereisis provides significant improvements in pain and function in appropriately selected paediatric patients while maintaining a favorable safety profile [10, 14].

Radiographic findings closely paralleled the observed clinical improvement. Both Kite's angle and Costa-Bertani angle improved significantly after surgery, and no significant changes were observed between the first- and third-year evaluations, indicating maintenance of the achieved correction over time. These findings are consistent with previous reports demonstrating durable restoration of hindfoot alignment after Calcaneo-stop arthroereisis [7, 11, 12]. The parallel improvement of radiographic parameters and validated clinical scores further supports the biomechanical rationale of the procedure, suggesting that restoration of physiological alignment translates into meaningful functional recovery.

The overall complication rate observed in the present series was low (7.4%). Most complications consisted of mild superficial wound erythaema that resolved completely following conservative treatment, whereas only two patients required revision surgery because of implant screw breakage. No cases of deep infection, neurovascular injury, implant loosening, overcorrection, or undercorrection were observed. These findings compare favorably with previous clinical studies and systematic reviews reporting low complication rates following Calcaneo-stop arthroereisis [10, 13–15]. In particular, the recent systematic review and meta-analysis by Pavone et al. reported an overall pooled complication rate of 7.97%, which is remarkably consistent with the 7.4% complication rate observed in our cohort. Although implant-related complications have been described in the literature, their incidence appears to remain limited, particularly in experienced centres.

An important aspect of the present investigation is that elective implant removal was not considered an outcome measure. According to our institutional protocol, screw removal may be considered approximately three to four years after surgery when clinically indicated. Since many patients had not yet reached this stage of treatment at the time of analysis, implant removal was intentionally excluded from the predefined study endpoints. Consequently, the present study specifically evaluates the medium-term clinical and radiographic effectiveness of Calcaneo-stop arthroereisis rather than long-term implant management.

Several methodological aspects strengthen the present study. Consecutive patient enrollment minimized selection bias, while standardized clinical and radiographic assessments ensured consistent follow-up evaluation. Moreover, bilateral procedures were analyzed at the patient level by averaging radiographic measurements from both feet, thereby avoiding pseudoreplication and unit-of-analysis bias. The use of validated functional outcome measures together with objective radiographic parameters further provided a comprehensive assessment of treatment effectiveness [4–8].

Nevertheless, several limitations should be acknowledged. First, despite prospective collection of clinical and radiographic data according to a standardized institutional protocol, the present investigation represents a retrospective analysis performed at a single institution. Second, no control group was available for comparison with alternative surgical procedures or continued conservative management. Third, body mass index was not routinely recorded and therefore could not be included in the exploratory multivariable analyses. Finally, although radiographic measurements were independently performed by three experienced orthopaedic surgeons using standardized techniques, formal assessment of interobserver reliability was not performed.

Despite these limitations, the present study includes a relatively large consecutive single-center cohort with standardized clinical and radiographic evaluation and a minimum follow-up of three years. The consistency between clinical improvement and radiographic correction, together with the low complication rate observed, provides additional evidence supporting Calcaneo-stop arthroereisis as an effective, reproducible, and safe treatment option for symptomatic pediatric flexible flatfoot.

## Conclusion

Calcaneo-stop arthroereisis resulted in significant clinical, functional, and radiographic improvement in children with symptomatic flexible flatfoot, with correction maintained at a minimum follow-up of three years. These findings support the medium-term effectiveness of the procedure in appropriately selected paediatric patients.

## Data Availability

No datasets were generated or analysed during the current study.
